# Evaluating factors influencing customers’ intention to eat Korean cuisine “Samgyeopsal” in the Philippines: A structural equation model forest classifier approach

**DOI:** 10.1371/journal.pone.0286077

**Published:** 2023-05-19

**Authors:** Ardvin Kester S. Ong, Yogi Tri Prasetyo, Atheena Rhezelle B. Manguray, E. J. Meinard G. Moral, Andrea Lorraine M. Maun, Josh Gasty F. Diaz, Charlotte N. Monteiro, Venice Cristine C. Dangaran, Satria Fadil Persada, Reny Nadlifatin, Irene Dyah Ayuwati

**Affiliations:** 1 School of Industrial Engineering and Engineering Management, Mapúa University, Manila, Philippines; 2 International Bachelor Program in Engineering, Yuan Ze University, Chung-Li, Taiwan; 3 Department of Industrial Engineering and Management, Yuan Ze University, Chung-Li, Taiwan; 4 Young Innovators Research Center, Mapúa University, Manila, Philippines; 5 Malayan High School of Sciences, Pandacan, Maynila, Philippines; 6 Entrepreneurship Department, BINUS Business School Undergraduate Program, Bina Nusantara University, Jakarta, Indonesia; 7 Department of Information Systems, Institut Teknologi Sepuluh Nopember, Surabaya, Indonesia; 8 Department of Information Systems, Institut Teknologi Telkom Surabaya, Surabaya, Indonesia; Wroclaw University of Environmental and Life Sciences: Uniwersytet Przyrodniczy we Wroclawiu, POLAND

## Abstract

Samgyeopsal has become a widely popular cuisine in the Philippines since 2014. The rise of Samgyeopsal is evident worldwide as it is available in countries such as the United States, Northern, and Southern Asia. This study aimed to explore the intention to eat Samgyeopsal during the COVID-19 pandemic utilizing structural equation modeling and random forest classifier. With a total of 1014 responses collected online, the result showed that utilitarian and hedonic motivation, Korean influence, and attitude led to very high actual behavior in east Samgyeopsal in the Philippines. Moreover, subjective norm, perceived behavioral control, and intention led to significant results influencing intention to actual behavior. Lastly, the COVID-19 safety protocol showed the least significant result. This study is the first study that evaluated the intention of consumers to eat Samgyeopsal in the Philippines during the COVID-19 pandemic. The results of this study would be beneficial to Korean BBQ restaurateurs and the further development of their marketing strategies even in other countries. Finally, the model construct of this study can be extended and applied in evaluating the consumers’ eating intention toward other varieties of food or cuisines worldwide.

## 1. Introduction

The Korean wave has had a great impact on the Philippines. It is also known as “Hallyu—wave or flow of Korea” where Korean films, Korean pop music (K-pop), and even Korean dramas have become popular [1, 2]. The Philippines had the highest growth rate of Hallyu enthusiasts among 113 nations [2]. As an impact, the media popularization of the Korean wave has increased its influence, even on the cuisines [3]. Eventually, the Filipinos’ consumption of these media has taken their level of interest in Korean cuisine [4].

Korean cuisine such as Korean barbeque (Korean BBQ), commonly referred to by Filipinos as Samgyeopsal, has rapidly become a popular social and dining activity in the Philippines [5–7]. According to a 2020 survey conducted by the Korea Trade-Investment Promotion Agency (KOTRA) in Manila, the number of Korean restaurants in the Philippines has climbed by 81.2 percent from 2014 to 2018. Consequently, all-you-can-eat buffets such as Korean BBQ restaurants are highly popular in the Philippines as they give great offers to consumers who are looking to eat as much as possible at an affordable price. Moreover, numerous restaurateurs have started franchises that market Samgyeopsal in different varieties.

**Fig 1:**
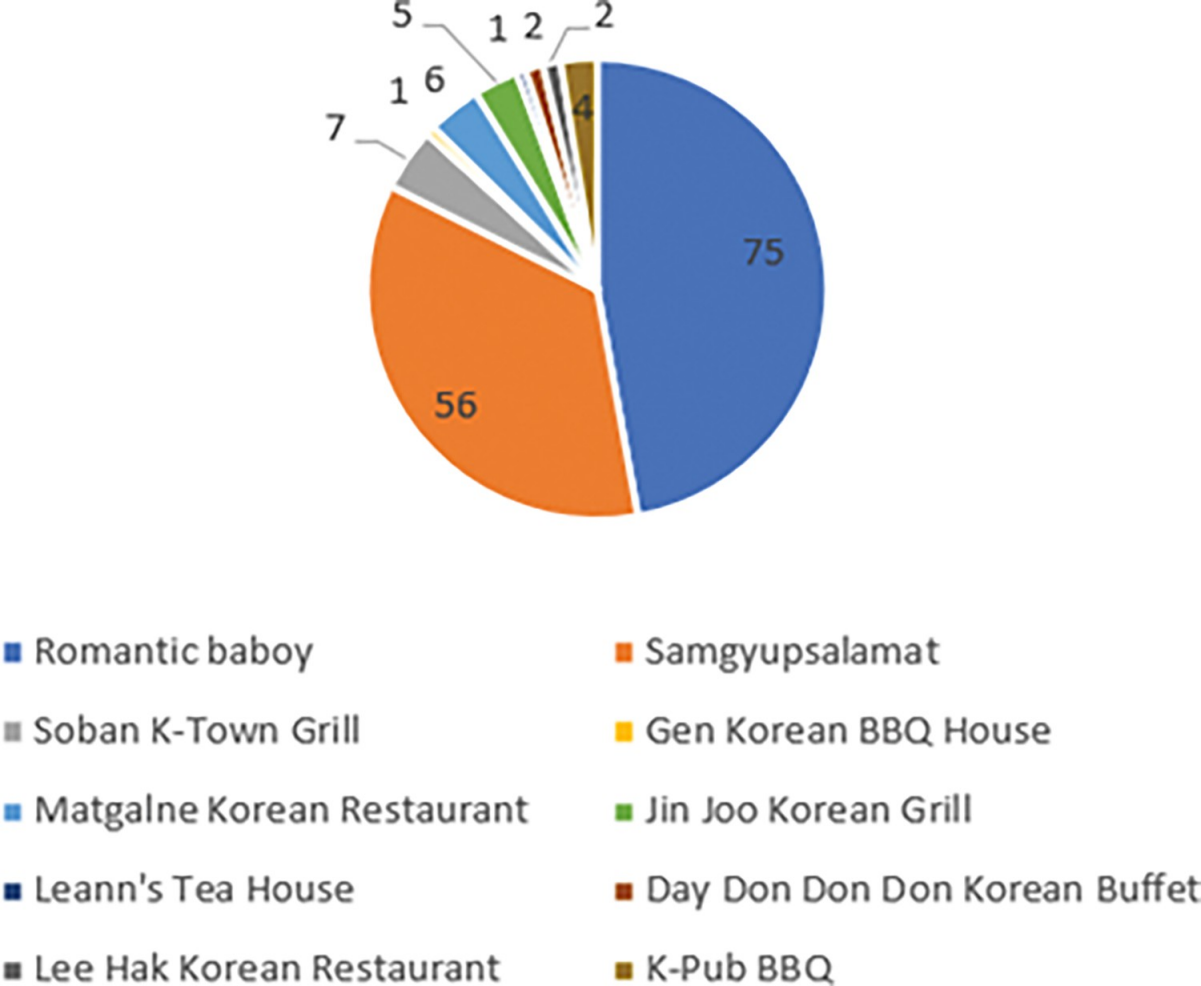
Top 10 Korean BBQ restaurants in the Philippines [5–7].

Fig 1 represents the top 10 Korean barbecue restaurants in the Philippines and their respective number of branches. Based on this figure, it can be observed that Romantic Baboy and Samgyupsalamat are the two restaurants that have the most number of branches in the Philippines. They have a combined number of 131 branches in the Philippines while the other restaurants range from 1 to 7. Ultimately, the figure below shows that the top 10 Korean BBQ restaurants currently have a total of 159 branches all over the Philippines [5–7].

Various studies on meat linked with Samgyeopsal will help provide better information on how research on meat or Korean barbecue was conducted. Nam et al. [8] studied Samgyeopsal relating to East Asian meat products and consumption habits. Their results showed that East Asia’s dynamic environment, religion, history, and main food staples have contributed to a lack of meat consumption culture. Recently, due to the globalization of the food business and the countries’ rapid economic expansion, the amount of meat produced and consumed in Eastern countries has increased dramatically. However, the study of Nam et al. [8] only focused on three Northeast Asian countries: China, Japan, and Korea, and two Southeast Asian countries, Vietnam and Thailand. Both Vietnam and Thailand have traditionally shared comparable environmental and cultural contacts but have their own distinct cuisine cultures.

Cultural influences lead to the variation in the practices of preparation and in the consumption of food and cuisine [9]. It influences shapes the fundamental structure of food attitudes [10]. The consumers’ experiences, beliefs, and values are shaped by culture, which is connected to attitudes, emotions, social norms, intentions, and behavior [11]. The impact of culture in correlation with food choice or consumption has been tackled by different studies [3, 10, 12] and is commonly measured using the Theory of Planned Behavior.

The current times changed the behavior of people [13, 14]. It was explained in the different studies how reassessment of behavioral factors should be considered. To which, their study implemented the utilization of TPB, SERVQUAL 5 dimension, and even the social exchange theory. The separate analysis made provided justification for the change in human behavior due to the influence that happened during the strict lockdown, health fear, and perception of people [15]. As well as their study focusing on the transportation sector, restaurants have also been influenced due to changes in the behavioral aspects of people. Thus, the need to evaluate behavioral intention through TPB is needed to further close the gap of changes that happened during the COVID-19 pandemic [16]. In accordance, the lack of study focusing on Samgyeopsal despite its popularity should be evaluated to provide managerial implications and suggestions for restaurants in the near end of the COVID-19 pandemic such as in the Philippines.

The Theory of Planned Behavior (TPB) is a commonly used expectancy-value model of attitude-behavior interactions that has been successful in predicting a range of behaviors to some extent [17–20]. According to the TPB, attitudes, subjective norm, and perceived behavioral control predicts behavioral intention which then affects the actual conduct of a person [21]. The model of the TPB is beneficial in predicting the behavioral intentions of individuals to predict consumers’ purchase intentions [18]. Several studies have used the TPB to determine the intention and measure human behavior [22–24]. However, only a few researchers have used the theory to investigate a consumer’s satisfaction and intention for eating meals [25, 26].

Hoeksma et al. [27] utilized an extended TPB to predict consumers’ willingness to purchase mobile slaughter unit (MSU) meat. Their results showed that consumers who decided to buy MSU meat have a stronger intention in buying. However, the study only covered pork and beef meat. Charlton et al. [28] considered a Visual Analogue Scale (VAS) rating for hunger and satiety over a set time. Pork, beef, and chicken were test meals used in this study. Their result showed that all three types of meats had similar effects on eating behavior. However, the study only covered three (3) meals to represent the differences in the release of intestinal hormones associated with appetite and hunger. Thus, studies on Samgyeopsal have been underexplored despite the consequent impact and rise of popularity with the Korean wave on Filipinos eating Korean food, particularly Samgyeopsal.

This study aimed to evaluate the varying factors influencing consumers’ intention to eat Samgyeopsal in the Philippines by extending the Theory of Planned Behavior. Various factors such as hedonic motivation, utilitarian, Korean influence, attitude, subjective norms, perceived behavioral control, COVID-19 safety protocols, intention, and actual behavior were analyzed using the Structural Equation Modelling (SEM) and Random Forest Classifier approach. This is considered the first study to analyze the different factors influencing consumers’ intention to eat Samgyeopsal in the Philippines during the COVID-19 delta variant pandemic. The results of this study would be beneficial to Korean BBQ restaurateurs and the further development of their marketing strategies since Samgyeopsal is a business that has been growing exponentially, even in other countries. Finally, the model construct of this study can be extended and applied in evaluating the consumers’ eating intention toward other varieties of food or cuisines.

## 2. Conceptual framework

**Fig 2:**
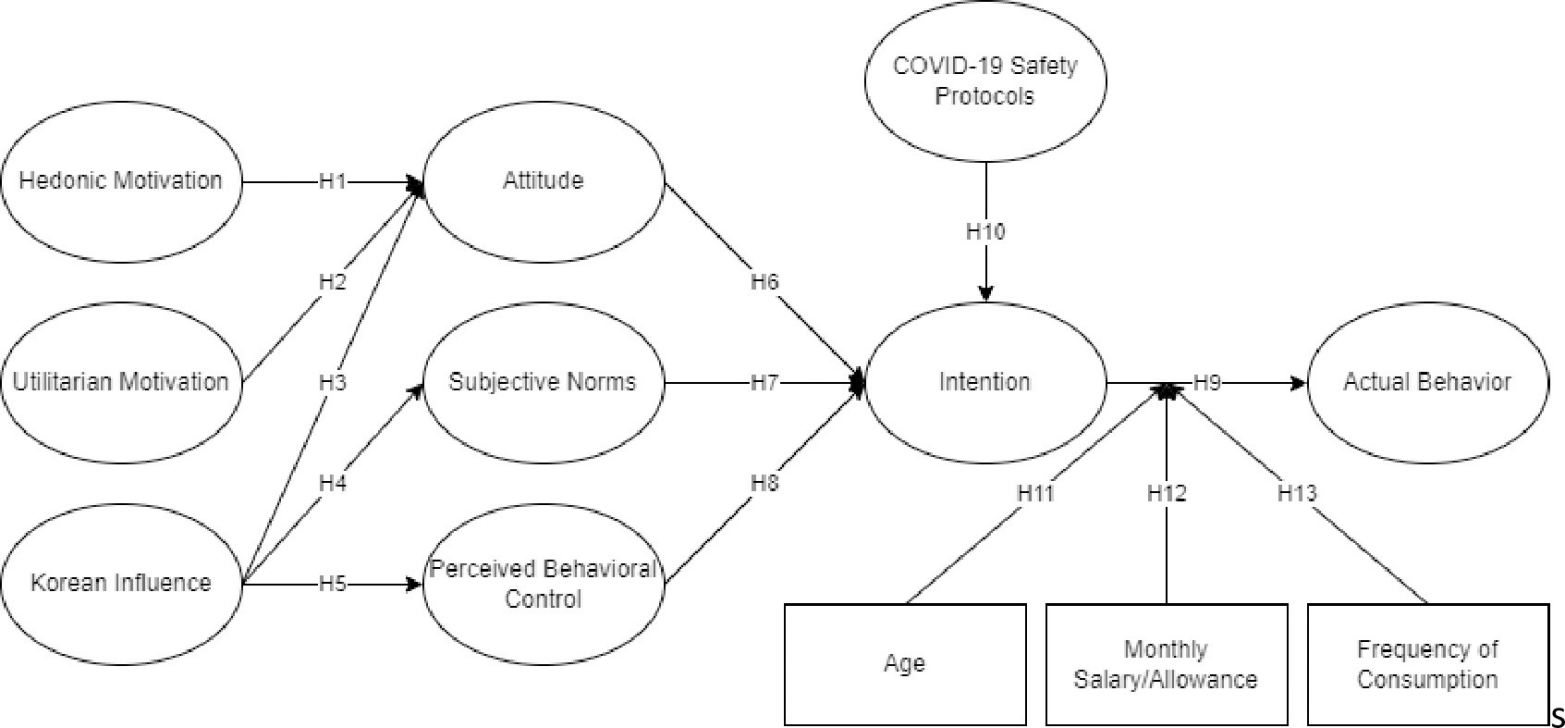
Conceptual framework.

This study considered the extended Theory of Planned Behavior (TPB) to evaluate factors affecting the influence of consumers’ intention to eat Samgyeopsal in the Philippines during the COVID-19 delta variant. As seen in Fig 2, there were 10 hypotheses created for this study that included different latent such as hedonic motivation, utilitarian, Korean influence, attitude, subjective norms, perceived behavioral control, COVID-19 safety protocols, intention, and actual behavior.

Hedonic motivation refers to a person’s willingness to engage in acts that improve positive experiences (pleasant or nice) while also reducing bad experiences [29]. The link between beliefs and attitudes is analogous to hedonic motivation [30]. Hedonic motivation has been discovered to have a significant impact on cognitive-emotional attitudes about purchasing intentions. Moon et al. [31] presented in their study how consumers’ perceptions of hedonic attributes are important and are considered a positive predictor of cognitive and affective attitude. Ong et al. [22] also presented how hedonic motivation had a positive significant direct effect on attitude of consumers. Thus, it was hypothesized that:

**H**_**1**_. Hedonic motivation had a significant direct positive effect on attitude.

The utilitarian aspect of a consumer’s attitude toward an action relates to the behavior’s utility, value, and wisdom in the consumer’s eyes [32]. Attitude is directly affected by the Utilitarian motivation [33, 34]. Prior studies found that utilitarian traits are concerned with more cognitive components of attitude when shopping or purchasing (*e*.*g*., convenience, economic value for money, or time savings) [35–37]. Similarly, Ong et al. [22] showed how utilitarian motivation had a significant effect on consumers’ attitude when purchasing. Hence, it was hypothesized that:

**H**_**2**_. Utilitarian had a significant direct positive effect on attitude.

Consumers’ attitudes are positively influenced by the attachment and affinity features of Korean trending dramas [38]. With that, Lee et al. [39] stated that consumption of Korean items would ultimately lead to a better understanding of Korean culture and appreciation for Korea. This demonstrates that people’s understanding and acceptance of culture are highly influenced by their level of contact with the environment, whether directly or indirectly. In addition, media plays a vital role in learning to embrace the culture of a foreign place through experience [39]. Thus, it was hypothesized that:

**H**_**3**_. Korean influence had a significant direct positive effect on attitude.
**H**_**4**_. Korean influence had a significant direct positive effect on subjective norms.
**H**_**5**_. Korean influence had a significant direct positive effect on perceived behavioral control.

Attitude is an antecedent of intention, as well as the degree to which an individual has a previous valuation of possible behavior concerning any purchasing situation [17]. TPB specifies that attitudes, subjective norms, and perceived behavioral control are the essential components of behavioral intent and actual health-related behavior [40]. The subjective assessment of the risks and advantages of the expected outcome, as well as the attitude about the chance that the behavior will have the desired effect that subsequently influence behavioral intentions [41].

Lau et al. [42] mentioned that the variables of behavioral intention are directly correlated to the factors derived from the TPB. In addition, Ong et al. [43] presented how attitude is one of the highest factors influencing Filipinos that affects their intention. According to Latimer and Martin-Ginis [44], perceptions of others’ views may influence intention, depending on how much the individual fears being judged. In the context of the TPB, it is plausible that fear of being judged badly by others moderates the path from social standards to fast food intents [44]. In addition, Research suggests that if consumers possess high perceived behavioral control towards purchasing organic food products, positive behavioral intentions will also be high [45–47]. Thus, it was hypothesized that:

**H**_**6**_. Attitude had a significant direct positive effect on intention.
**H**_**7**_. Subjective norms had a significant direct positive effect on intention.
**H**_**8**_. Perceived behavioral control had a significant direct positive effect on intention.
**H**_**9**_. Intention had a significant direct positive effect on actual behavior.

Restaurants, where cluster infections are easily spread, have been a source of concern during the pandemic. Droplets, airborne particles, and person-to-person contact can disseminate COVID-19 and someone with the coronavirus infecting the environment and anything in it can easily cause its transmission [48]. Because of this, the people have become cautious and thus, influenced their intention to dine out and eat. To promote safe dining out, the government announced that restaurants must implement social distancing rules and set safety guidelines [7]. To reduce the risk of infection, close physical contact and food sharing in restaurants should be avoided. Other precautionary measures that restaurants can take to make customers feel safer and reduce physical and psychological risks include hand sanitizer, contactless payment, segregated dining areas, and separate restrooms. Hence, it was hypothesized that:

**H**_**10**_. COVID-19 safety protocols had a significant direct positive effect on intention.

As control variables, this study also considered demographic factors such as age, frequency of consumption, and monthly allowance/salary which may affect the actual behavior for consumption as presented among several studies [21, 29, 31, 34]. Based on the described presentation of the different studies, demographic characteristics may play a significant role in the actual behavior of consumers from a different perspective. In addition, the presentation of age, income or allowances, and frequency had presented a great factor among consumption or actual behavior on a positive or negative decision [22, 30]. Therefore, the following were hypothesized:

**H**_**11**_. Age as a control variable had a significant direct positive effect on actual behavior.
**H**_**12**_. Monthly Allowance/Salary as a control variable had a significant direct positive effect on actual behavior.
**H**_**13**_. Frequency of consumption as a control variable had a significant direct positive effect on actual behavior.

## 3. Methodology

### 3.1 Participants

The study employed a self-administered online cross-sectional survey using Google forms. Due to the COVID-19 pandemic, the survey was distributed online via social media platforms [49]. This study used a convenience sampling approach, and respondents were asked to sign a consent form before answering the study’s questionnaire. As shown in Table 1, a total of 1,014 people voluntarily responded to the survey. This study underwent verification by Mapua University Research Ethics Committees. Informed consent was obtained from all participants prior to the data collection in accordance with Data Privacy Act or Republic Act No. 10173 in the Philippines. Utilizing the Yamane Taro equation, the Philippines’ total population is 62.6 million. At 95% confidence, 399 or roughly 400 respondents would suffice the generalizability [50]. The current study had 1014 respondents, which may be confidently a representation of the consumers.

**Table 1:** The demographic of the respondents (n = 1014).

Characteristics	Category	n	%
Gender	Male	433	42.7
	Female	557	54.9
	Other	24	2.40
Age	15–24	903	89.1
	25–34	29	2.90
	35–44	57	5.60
	45–54	17	1.70
	Above 54	8	0.80
Monthly Salary/Allowance	<15,000 PHP	820	80.9
	15,000–30,000 PHP	89	8.80
	30,001–45,000 PHP	40	3.90
	45,001–60,000 PHP	22	2.20
	60,001–75,000 PHP	11	1.10
	>75,000 PHP	32	3.20
How many times do you eat Samgyeopsal in a month?	1	697	68.7
	2	216	21.3
	3	66	6.50
	4	10	1.00
	5	5	0.50
	more than 5	20	2.00
Location	Region I	45	4.10
	Region II	6	0.60
	Region III	120	11.8
	Region IV-A	234	23.1
	Region IV-B	6	0.60
	Region V	19	1.90
	CAR	8	0.80
	NCR	465	45.9
	Region VI	16	1.60
	Region VII	17	1.70
	Region VIII	4	0.40
	Region IX	18	1.80
	Region X	5	0.50
	Region XI	22	2.20
	Region XII	8	0.80
	Region XIII	8	0.80
	BARMM	16	1.60

The demographics are made up of 433 males, 557 females, and 24 other genders with the majority belonging from the 15 to 24 years old age bracket (85.40%). Furthermore, approximately 80.87% receive a monthly salary/allowance of <15,000 PHP, and eat Samgyeopsal once a month (68.74%). Most of the respondents came from National Capital Region (45.86%). The result is supported by Gallinera et al. [51] who indicated that the majority of the consumers of Samgyeopsal in the Philippines have low budgets and are between 15–35 years old.

### 3.2 Questionnaire

Table 2 presents the constructs that were used to create this study’s questionnaire that can assess the various factors influencing consumers’ intentions to eat Samgyeopsal or Korean BBQ in the Philippines. Following the study of Ong et al. [22, 43], a 5-point Likert scale was used to measure all of the latent constructs using Structural Equation Modelling (SEM). The questionnaire consisted of eleven sections: (1) Consent of the Respondents (2) Demographic Information (gender, age, monthly salary/allowance, and health insurance). (3) Hedonic Motivation, (4) Utilitarian, (5) Korean Influence, (6) Attitude, (7) Subjective Norms, (8) Perceived Behavioral Control, (9) COVID-19 Safety Protocols, (10) Intention, and (11) Actual Behavior. A total of 54 questions were developed for this study and adapted from different studies. Prior to the distribution and apart from the approval of the Ethics Committee, the questionnaire was subjected to preliminary assessment considering 150 respondents. The overall results showed acceptable findings (Cronbach’s alpha > 0.70, Hair [52]). To which, the full questionnaire was utilized for distribution and data collection.

**Table 2:** The constructs and measurement items.

Constructs	Items	Measures	References
Hedonic Motivation	HM1	I feel better when I eat Samgyeopsal.	Taquet et al. [53]
	HM2	I enjoy eating Samgyeopsal.	Ajzen [18], Lam & Hsu [54]
	HM3	I find pleasure in eating Samgyeopsal.	Ajzen [18], Lam & Hsu [54]
	HM4	Eating Samgyeopsal makes me excited.	López et al. [55] Dhar & Wertenbroch [56]
	HM5	I feel happy when I eat Samgyeopsal.	
Utilitarian	U1	I can’t live without Samgyeopsal.	Dhar & Wertenbroch [56]
	U2	I feel accomplished after eating Samgyeopsal.	Childers et al. [57]
	U3	Eating Samgyeopsal satisfies my needs.	Dhar & Wertenbroch [56]
	U4	I think eating Samgyeopsal is worth it.	Zeithaml [37]
	U5	Eating Samgyeopsal is worth my time.	Izquierdo-Yusta et al. [58]
Korean Influence	KI1	Korean dramas influenced me to eat Samgyeopsal.	Jang & Paik [59]
	KI2	Korean dramas introduced me to Samgyeopsal.	Jang & Paik [59]
	KI3	I eat Samgyeopsal because of the Korean idols I idolize.	Jang & Paik [59]
	KI4	I eat Samgyeopsal because I am fond of Korean culture.	Jang et al. [60]
	KI5	Korean Culture influenced me to eat Samgyeopsal.	Jang et al. [60]
Attitude	A1	I feel like eating samgyeopsal is a good choice	Huang & Ge [61], Wang et al. [62]
	A2	I like the samgyeopsal restaurant that I frequently visit	Ajzen [18]
	A3	Eating samgyeopsal uplifts my mood	Ajzen [18], Lam & Hsu [54]
	A4	The experience in eating samgyeopsal is good that is why I like it.	Choe & Kim, [63]
	A5	I feel that eating Samgyeopsal helps me relieve stress	Choe & Kim, [63]
Subjective Norms	SN1	The trend of eating Samgyeopsal among people around me is increasing.	Al-Swidi et al. [45]
	SN2	Most people I know have eaten Samgyeopsal.	Ajzen [18], Lam & Hsu [54]
	SN3	Most people I know think that I should eat Samgyeopsal.	
	SN4	Most people I know approve me eating Samgyeopsal.	
	SN5	People whose opinions I value would prefer that I eat Samgyeopsal.	Youn et al. [64]
	SN6	My friends prefer to eat Samgyeopsal over other food.	
	SN7	My friends always choose to dine in Samgyeopsal restaurants whenever we go out.	
	SN8	My family prefers to eat Samgyeopsal over other food.	
	SN9	My family always chooses to dine in Samgyeopsal restaurants whenever we go out.	
Perceived Behavioral Control	PBC1	It’s up to me whether to eat Samgyeopsal or not.	Kim & Kim [65]
	PBC2	I can go to a Samgyeopsal restaurant whenever I want.	
	PBC3	I have the financial capability to dine in a Samgyeopsal restaurant.	
	PBC4	Eating Samgyeopsal in the future is up to me.	Ajzen [18], Lam & Hsu [54]
	PBC5	I am confident that I can purchase Samgyeopsal in the future.	
COVID-19 Safety Protocols	CSP1	I practice social distancing when eating in a Samgyeopsal restaurant or having the food delivered.	FDA [66]
	CSP2	I wash my hands with soap and water for 20 seconds before eating Samgyeopsal.	FDA [66]
	CSP3	The establishment frequently cleans the floors, counters, and other facility access areas.	FDA [66]
	CSP4	The employees use gloves to avoid direct bare hand contact with the food.	FDA [66]
	CSP5	The establishment frequently sanitizes their equipment and utensils.	FDA [66]
	CSP6	The establishment frequently sanitizes the surfaces touched by the customers or employees (doorknobs, equipment handles, & check-out counters)	FDA [66]
	CSP7	I pay using my phone or via online to avoid the physical transfer of cash which can potentially carry the virus.	Sanew [67]
	CSP8	I transfer the food out of the packaging and dispose of it.	FDA [66]
	CSP9	I clean the area where I place the bag/packaging.	FDA [66]
Intention	I1	I intend to continue to eat Samgyeopsal.	Kim & Kim [65]
	I2	I will tell others positively about eating Samgyeopsal.	Kim & Kim [65]
	I3	I want to eat Samgyeopsal food in the future.	Ajzen [18], Lam & Hsu [54], Zeithaml et al. [37]
	I4	I intend to eat Samgyeopsal food in the future.	Ajzen [18], Lam & Hsu [54], Zeithaml et al. [37]
	I5	I would also recommend others to eat Samgyeopsal.	Al-Swidi et al. [45]
Actual Behavior	AB1	I treat/reward myself with Samgyeopsal.	Dunn et al. [68]
	AB2	I eat Samgyeopsal as I cannot cook.	Dunn et al. [68]
	AB3	I eat Samgyeopsal even if I’m alone.	Dunn et al. [68]
	AB4	I eat Samgyeopsal when I crave for beef or pork.	Coşkun & Özbük [69]
	AB5	I plan to eat as much as possible because of the cost.	Coşkun & Özbük [69]
	AB6	Whenever I have thoughts about food, I automatically think about eating Samgyeopsal.	Ajzen [18], Lam & Hsu [54], Zeithaml et al. [37]

### 3.3 Structural equation modeling

Structural Equation Modeling (SEM) is a combination of statistical techniques for calculating and analyzing the correlations between observable and latent variables. It investigates linear causal links between variables while taking measurement error into account [70]. The correlations between grandparenting practices, food insecurity, and childhood depression were investigated using SEM [71]. Cunha et al. [72] conducted a study on the differences between observed and self-reported food safety practices by using SEM since it compensates for the model’s complexity and may corroborate the distinction between observed and self-reported behaviors. SEM investigates the possible mechanisms through which information and perceptions influence behavior [73]. Hence, this study utilized SEM to evaluate the factors that influence consumers’ intention to eat Samgyeopsal in the Philippines by utilizing the extended theory of planned behavior.

### 3.4 Random Forest Classifier

Random Forest Classifier (RFC) is a type of decision tree that produces higher accuracy in classifying factors [74]. This type of algorithm has several advantages such as easy interpretation and data processing. Moreover, the interpretation of the result is also considered to be uncomplicated [75]. Different studies regarding human behavior utilized RFC to classify the different factors considered [74, 76, 77]. Compared to other machine learning tools, the RFC provides a higher accuracy rate and easier optimization, processing, and analysis. It was also established that RFC shows only the highly significant factors affecting a subject matter. German et al. [50] explained that mostly, RFC can suffice as support for SEM analysis since it provides insight into the significant latent variables relating to the dependent variables which may be significant due to the relative indirect effects but may not be evident.

Similarly, German et al. [50] showed the accuracy rates of different machine learning tools. To which, neural networks may provide better accuracy rates, but would have higher standard deviation compared to RFC which shows the consistent output. In addition, Chen et al. [74] expounded on the efficiency compared to other advanced machine learning tools that may also provide higher accuracy rates, but are more complicated those present almost similar findings. Thus, this study considered the utilization of RFC to predict factors that significantly affect the actual behaviour of consumers in eating Samgyeopsal in the Philippines.

Before running the RFC, data cleaning using correlation analysis was done for the 1014 responses collected. Only significant values (p-value < 0.05) and high correlation (> 0.20) were considered for the 54,756 data. Following this was data aggregation and data normalization. In utilizing RFC, different training and testing ratio (60:40, 70:30, 80:20, 90:10) were utilized. To which, criterion (gini and entropy) and splitter (best and random) were utilized following the study of Yang and Zhou [77]. A total of 9,600 runs were done for the optimization process using Python 3.8.

## 4. Results

**Fig 3:**
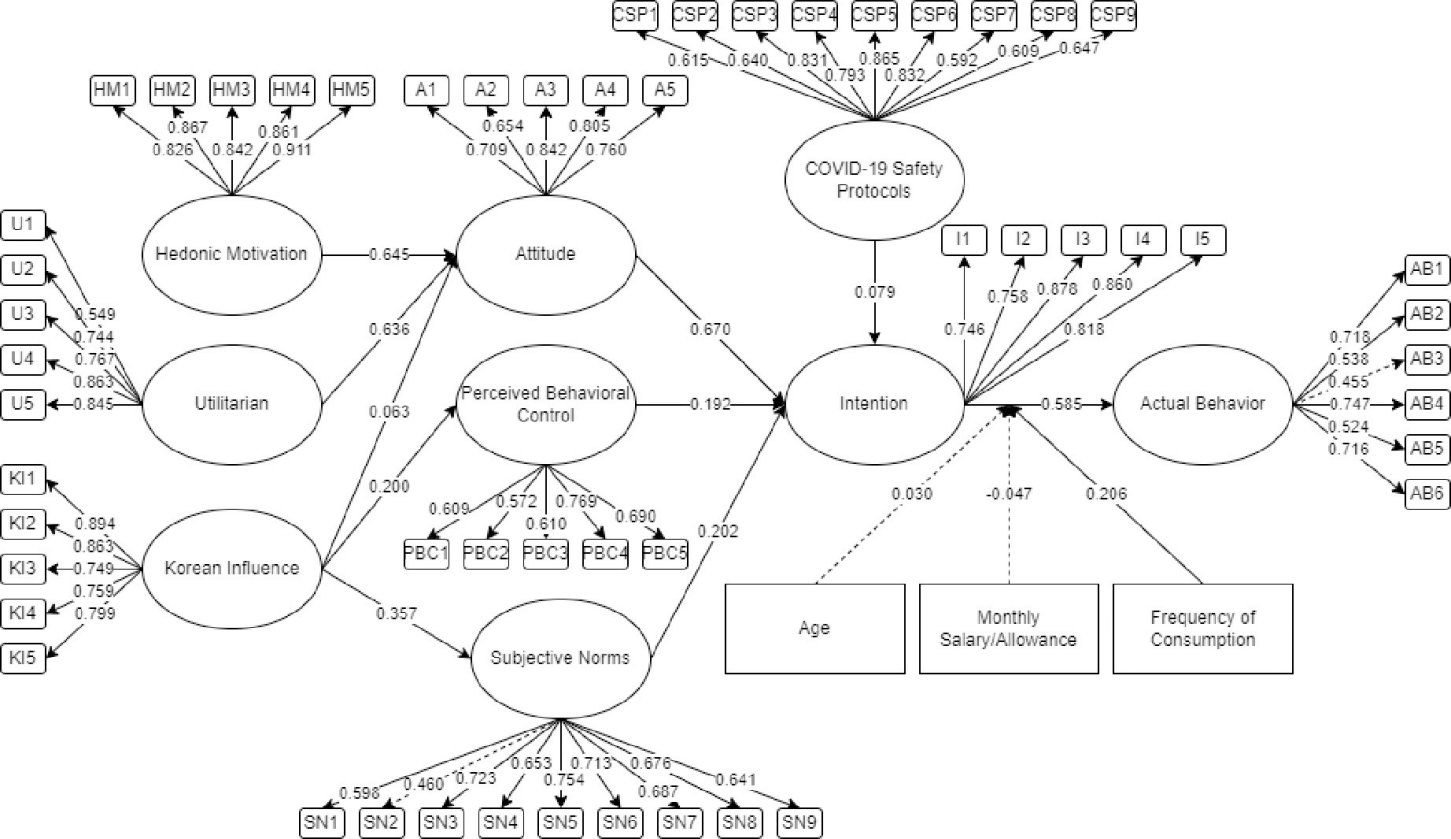
The initial SEM for consumers’ intention to eat Samgyeopsal.

Fig 3 represents the initial SEM for consumers’ intention to eat Samgyeopsal in the Philippines. Utilizing AMOS 25 for a factor-based model, the indicators were visually inspected for values below the 0.5 threshold. According to Ong et al. [49] and Hair [52], the removal of non-significant latent and indicators below 0.5 may be done to increase the model fit. As seen in Fig 3, SN2 and AB3 were below 0.5, therefore, were removed.

**Fig 4:**
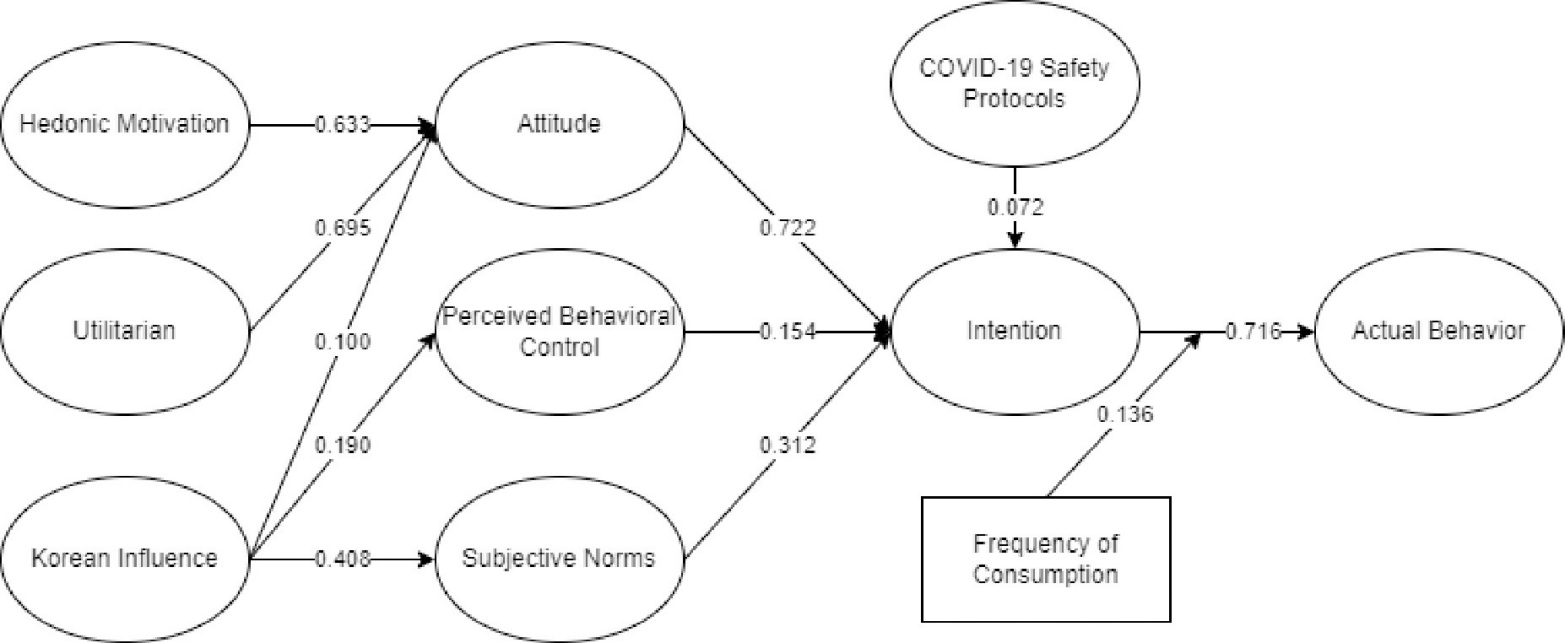
The final SEM for consumers’ intention to eat Samgyeopsal.

From the initial SEM, the latent were all significant. Fig 4 represents the final SEM for consumers’ intention to eat Samgyeopsal in the Philippines with significant latent variables and indicators. Presented in Table 3 are the descriptive statistics of the factor loading. It could be seen that the final factor loading had values greater than 0.50.

**Table 3:** Indicators statistical analysis.

Variable	Item	Mean	StD	Factor Loading	
				Initial	Final
Hedonic Motivation	HM1	4.107	0.984	0.826	0.843
	HM2	4.502	0.831	0.867	0.877
	HM3	4.214	0.983	0.842	0.850
	HM4	4.272	0.993	0.861	0.845
	HM5	4.378	0.903	0.911	0.906
Utilitarian	U1	2.108	1.175	0.549	0.877
	U2	3.351	1.187	0.744	0.698
	U3	3.232	1.180	0.767	0.728
	U4	3.969	1.080	0.863	0.858
	U5	3.989	1.073	0.845	0.848
Korean Influence	KI1	3.046	1.591	0.894	0.806
	KI2	2.970	1.649	0.863	0.663
	KI3	2.176	1.377	0.749	0.776
	KI4	2.901	1.416	0.759	0.847
	KI5	3.038	1.453	0.799	0.889
Subjective Norm	SN1	4.119	1.009	0.598	0.596
	SN2	4.390	0.907	0.460	-
	SN3	3.623	1.171	0.723	0.796
	SN4	4.070	1.009	0.653	0.753
	SN5	3.532	1.148	0.754	0.823
	SN6	3.094	1.208	0.713	0.607
	SN7	2.990	1.289	0.687	0.885
	SN8	2.479	1.220	0.676	0.899
	SN9	2.434	1.259	0.641	0.843
Attitude	A1	3.840	1.040	0.709	0.725
	A2	3.841	1.065	0.654	0.638
	A3	4.035	1.047	0.842	0.798
	A4	4.033	1.030	0.805	0.768
	A5	3.918	1.118	0.760	0.728
Perceived Behavioral Control	PBC1	4.277	1.019	0.609	0.609
	PBC2	3.350	1.345	0.572	0.659
	PBC3	3.587	1.205	0.610	0.865
	PBC4	4.281	0.977	0.769	0.880
	PBC5	4.164	1.057	0.690	0.627
COVID-19 Safety Protocol	CSP1	4.570	0.827	0.615	0.682
	CSP2	4.532	0.830	0.640	0.690
	CSP3	4.358	0.720	0.831	0.817
	CSP4	4.349	0.912	0.793	0.805
	CSP5	4.434	0.844	0.865	0.881
	CSP6	4.264	0.922	0.832	0.842
	CSP7	4.177	1.098	0.592	0.569
	CSP8	4.156	1.040	0.609	0.585
	CSP9	4.385	0.931	0.647	0.619
Intention	I1	4.058	1.031	0.746	0.742
	I2	4.095	0.969	0.758	0.730
	I3	4.287	0.959	0.878	0.743
	I4	4.240	0.992	0.860	0.736
	I5	4.273	0.951	0.818	0.761
Actual Behavior	AB1	3.734	1.265	0.718	0.804
	AB2	2.441	1.308	0.538	0.873
	AB3	2.979	1.521	0.455	-
	AB4	3.340	1.381	0.747	0.589
	AB5	3.725	1.288	0.524	0.576
	AB6	2.755	1.365	0.716	0.761

Table 4 represents the parameters for model fit. The threshold for the acceptable range was adopted from different studies [78, 79]. The minimum cut-off value is greater than 0.80 for IFI, TLI, CFI, GFI, and AGFI [78] and less than 0.07 for RMSEA [79]. From which, the different values were greater than 0.80 and RMSEA presented 0.058. Ong et al. [22] and Hair [52] stated that when the model fit parameters were within range, then the SEM is considered acceptable.

**Table 4:** The model fit.

Goodness of fit measures of SEM	Parameter Estimates	Minimum cut-off	Suggested by
Incremental Fit Index (IFI)	0.896	>0.80	Gefen et al. [78]
Tucker Lewis Index (TLI)	0.884	>0.80	Gefen et al. [78]
Comparative Fit Index (CFI)	0.896	>0.80	Gefen et al. [78]
Goodness of Fit Index (GFI)	0.814	>0.80	Gefen et al. [78]
Adjusted Goodness of Fit Index (AGFI)	0.823	>0.80	Gefen et al. [78]
Root Mean Square Error (RMSEA)	0.058	<0.07	Steiger [79]

The composite reliability (CR), average variance extracted (AVE), and Cronbach’s alpha are presented in Table 5 for the test of the internal validity and reliability of the constructs. Hair [52] stated that the values of CR and Cronbach’s alpha should be greater than 0.70, while the AVE should be greater than 0.50 to present validity and reliability among the constructs. Lastly, setting an acceptable Variance Inflation Factor (VIF) value of 5, all the variables were seen to be below the threshold which presents further acceptability. As seen in Table 5, all the latent possessed values greater than the threshold which indicates internal validity and reliability.

**Table 5:** Composite reliability and validity.

Factor	Cronbach’s α	Composite Reliability (CR)	Average Variance Extracted (AVE)	Variance Inflation Factor (VIF)
Hedonic Motivation	0.933	0.937	0.747	3.447
Utilitarian	0.872	0.901	0.648	3.127
Korean Influence	0.908	0.898	0.640	1.197
Subjective Norm	0.865	0.926	0.613	4.841
Attitude	0.912	0.853	0.538	2.012
Perceived Behavioral Control	0.780	0.853	0.544	1.394
COVID-19 Safety Protocol	0.904	0.909	0.532	1.381
Intention	0.941	0.860	0.551	3.034
Actual Behavior	0.815	0.848	0.533	-

The Common Method Bias (CMB) was run using SPSS 25. The CMB utilizing Harman’s Single Factor should be below 50% to determine any bias among the constructs [43]. From the results, it was seen that the CMB had a 35.505% value, which is considered acceptable. Moreover, Garger et al. [80] that when CMB is not present, no single source bias would be evident. Lastly, Table 6 presents the direct, indirect, and total effects of the model. It could be seen that all hypotheses are significant.

**Table 6:** Direct, indirect, and total effects.

No	Variable	Direct Effect	P-Value	Indirect Effect	P-Value	Total Effect	P-Value
1	HM → A	0.633	0.001	-	-	0.633	0.001
2	U → A	0.695	0.003	-	-	0.695	0.003
3	KI → A	0.100	0.002	-	-	0.100	0.002
4	KI → SN	0.408	0.001	-	-	0.408	0.001
5	KI → PBC	0.190	0.002	-	-	0.190	0.002
6	A → I	0.722	0.002	-	-	0.722	0.002
7	SN → I	0.312	0.003	-	-	0.312	0.003
8	PBC → I	0.154	0.002	-	-	0.154	0.002
9	I → AB	0.716	0.003	-	-	0.716	0.003
10	CSP → I	0.072	0.049	-	-	0.072	0.049
11	HM → I	-	-	0.457	0.001	0.457	0.001
12	HM → AB	-	-	0.327	0.001	0.327	0.001
13	U → I	-	-	0.503	0.002	0.503	0.002
14	U → AB	-	-	0.359	0.002	0.359	0.002
15	KI → I	-	-	0.229	0.002	0.229	0.002
16	KI → AB	-	-	0.164	0.002	0.164	0.002
17	A → AB	-	-	0.517	0.002	0.517	0.002
18	SN → AB	-	-	0.224	0.003	0.224	0.003
19	PBC → AB	-	-	0.110	0.001	0.110	0.001
20	CSP → AB	-	-	0.052	0.048	0.052	0.048
21	Freq → AB	0.136	-	-	-	0.136	0.001

The summary of 9,600 runs of RFC results is presented in Table 7. Analyzing using Analysis of Variance, the 92% accuracy of prediction from ‘gini’ criterion and ‘best’ splitter showed the optimum result. Similar to the results of Yang and Zhou [77]. Utilizing the 80:20 training and testing ratio, the result showed a 0.00 standard deviation. High accuracy of 92% is considered to be acceptable and could be utilized for classification on predicting the actual behavior to eat Samgyeopsal in the Philippines [74].

**Table 7:** Decision tree mean accuracy (Depth = 5).

Category	60:40	70:30	80:20	90:10
**Random**				
**Gini**	82.22	81.08	84.33	83.70
**Std. Dev**	5.264	5.446	5.949	7.577
**Entropy**	82.50	81.91	83.71	85.38
**Std. Dev**	4.324	4.469	5.595	5.350
**Best**				
**Gini**	84.00	83.00	**92.00**	88.00
**Std. Dev**	0.000	0.000	**0.000**	0.000
**Entropy**	83.61	86.61	89.00	88.00
**Std. Dev**	0.492	0.492	0.000	0.000

**Fig 5:**
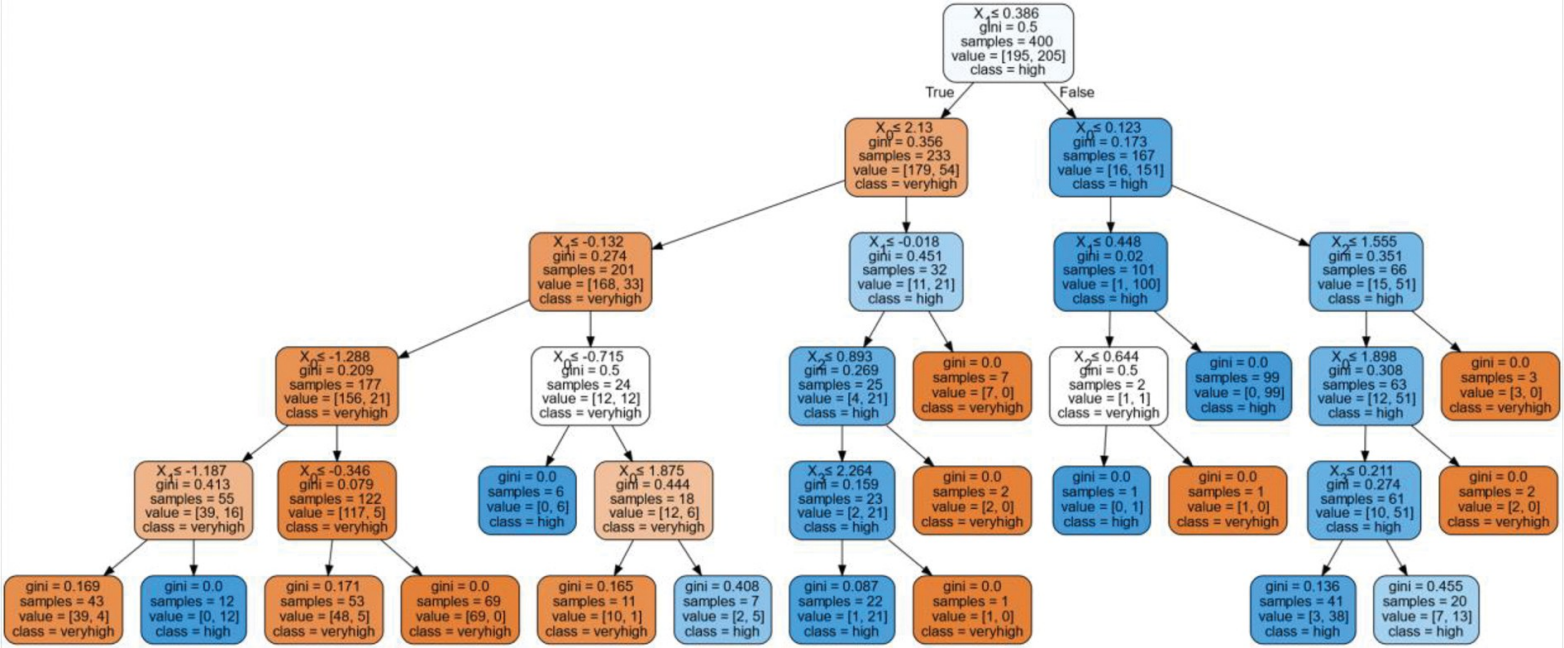
Optimum Random Forest Classifier.

Fig 5 presents the Optimum Decision Tree by Random Forest Classifier. It could be deduced that utilitarian motivation (X_1_) predicts the actual behavior of east Samgyeopsal in the Philippines. X_1_ would lead to hedonic motivation (X_0_) which would consider X_1_ or Korean influence (X_2_). If X_1_ was satisfied, it would also consider attitude which would lead to very high actual behavior to eat Samgyeopsal in the Philippines. If X_2_ would be considered, it will lead to high actual behavior to eat Samgyeopsal in the Philippines. This dictates that both utilitarian and hedonic motivation together with Korean influence and attitude predicts high to very high actual behavior to eat Samgyeopsal in the Philippines during the COVID-19 pandemic.

## 5. Discussion

Over the past years, Samgyeopsal has been one of the most popular social and dining activities in the Philippines. In this study, the SEM tool and RFC were utilized to examine the causal relationships between hedonic motivation (HM), utilitarian (U), Korean influence (KI), attitude (A), subjective norms (SN), perceived behavioral control (PBC), COVID-19 safety protocols (CSP), intention (I), and actual behavior (AB) in eating Samgyeopsal in the Philippines. An online survey was administered for this study with a total of 1,014 respondents.

The SEM results, it indicated that A had the highest significant direct effect on I (β = 0.722; p = 0.002). Based on the indicators, it was seen that Samgyeopsal restaurants were the primary choice among consumers, eating at Samgyeopsal lifted a person’s mood, there is a sense of relief, and there was an overall great experience when dining. Dunn et al. [68] suggested that positive moods and emotions may be orthogonal to their negative counterparts. This may be a critical factor to consider when recognizing that people may experience uncertainty within the affective component of their attitudes towards meals. This supports the significance of A to I. Emotions when eating Samgyeopsal were identified as major factors that influenced the intention to eat Samgyeopsal. This is found to be consistent with previous research suggesting that people’s attitudes have a major impact on their behavioral intentions [81].

Second, HM was found to have a significant effect on A (β = 0.633 and p = 0.001). Several key indicators under HM include feeling better, feeling enjoyment, pleasure, excitement, and happiness after eating Samgyeopsal which led to a positive A. These findings were supported by Ettis and Haddad [82] who also mentioned that HM is the key toenhancing the positive A. Similarly, Schuitema et al. [83] also highlighted the importance of HM towards positive A. It shows that positive A is linked to a higher expectation of hedonic incentives, according to other supporting studies.

Subsequently, UM was also found to have a significant effect on A (β = 0.695 and p = 0.003). After consuming Samgyeopsal, several signs related to UM, such as satisfying the needs, accomplishment, and functionality, were discovered to be crucial markers that contributed to a positive attitude. Ong et al. [22] backed up these findings, saying that UM is the key to improving one’s good attitude. Similarly, Aderonke [84] emphasized the significance of utility towards A. It demonstrates that A and perceived utility have a consistent and significant relationship with each other.

Fourth, I was found to have a significant direct effect to AB (β = 0.716; p = 0.003). Multiple factors under I such as continuing to eat Samgyeopsal, wanting to eat Samgyeopsal in the future, and recommending Samgyeopsal to others were identified as key indicators that resulted in positive AB. These findings were supported by Prasetyo et al. [85] who mentioned that people’s willingness to follow leads to healthy behavior. In addition, Eren and Gauld [86] specified the importance of I since it was found to be the only significant predictor of AB.

Fifth, KI had a significant direct effect on SN (β = 0.408; p = 0.001) and PBC (β: 0.190; p = 0.002). Indicators such as the influence of Korean trendy dramas, pop music, and Korean culture emphasized that the consumption of these media certainly influences a customer’s decision-making process. Moreover, KI shows to have a significant indirect effect to I (β = 0.229, p = 0.002) and AB (β = 0.164, p = 0.002). This finding suggests that KI is one of the most important considerations for I and AB. Thus, PBC (β = 0.229, p = 0.002), SN (β = 0.229, p = 0.002), and A (β = 0.229, p = 0.002) also led to having an indirect significant effect to AB.

Similar to the RFC results, HM, UV, A, and KI presented as significant factors that influenced actual behavior to eat Samgyeopsal in the Philippines. It could be deferred that the feeling of happiness, enjoyment, and functionality of eating Samgyeopsal would lead to a positive AB. Moreover, the influence of Korean pop, culture, and drama led to positive AB. These influences would have a positive attitude among consumers when it comes to the overall great experience, leaving them to continuously consume Samgyeopsal despite the COVID-19 pandemic.

Consequently, the SEM results also showed that SN had a significant direct effect on I (β = 0.312; p = 0.003). This indicates that the increasing trend of eating Samgyeopsal and the preference of the people surrounding them greatly impact their overall intention to eat Samgyeopsal as well. These findings have been corroborated by Jain [87] wherein it was discussed how SN and PBC were positively linked to luxury purchase intentions. Likewise, the results in the study of Ong et al. [7] showed that SN is the key motivator of purchase intention in relation to foods.

It was also found that PBC had significant effects on I (β = 0.154; p = 0.002). It implies that factors such as perceived confidence and financial capability are key predictors which lead to one’s definite intention to eat Samgyeopsal. The findings were consistent with previous research, which found that Koreans make extensive efforts to experience Yak-sun food, emphasizing that PBC is a significant and the strongest predictor of consumers’ behavioral intention to buy Yak-sun food [88]. Accordingly, Ong et al. [7] highlighted the significance of PBC as a more significant predictor of behavioral intention than any other construct.

Lastly, using the demographic factors as latent variables, only the frequency of Samgyeopsal consumption was seen to be significant. Other demographic factors were insignificant (p-value > 0.05). The frequency showed a significant direct effect on AB (β = 0.136; p = 0.001). Di Crosta et al. [89] explained that the frequency of consumption, especially in the pandemic era negates purchasing intention and the actual behavior of consumers. Most of which spend only on necessities. However, their results also explained that the consumption of unnecessary products would still depend on their economic capabilities. Rodrigues et al. [90] presented how demographic factors affect impulse consumption behavior, but when it comes to food consumption such as Samgyeopsal, this study has presented that age and monthly salary/allowance had no significant differences. From the descriptive statistics results, most of the consumers eat Samgyeopsal at least once (68.7%), twice (21.3%), or thrice (6.50%) a week. Despite the constant consumption, Liu [91] expounded on a possible cutback on indulgence. Thus, marketers may strategize on this effect by changing up schemes for Samgyeopsal consumption to reduce distaste for eating among consumers.

### 5.1 Recommendation

The extended TPB utilized in this study considered factors such as hedonic and utilitarian motivation, COVID-19 safety protocol, and Korean influence. Based on the results, all the latent considered were deemed to be significant. This study presents that the cultural influence and motivations have a significant effect on consumers’ intention to eat in Samgyeopsal restaurants. The framework utilized may be considered in other cuisine and restaurant that have been influenced by other cultures. In addition, the utilization of RFC showed high accuracy of classification to predict factors that greatly influence different latent. Following the discussion of Fan et al. [92], some factors may have lower significance due to the causal relationship present in the SEM. Thus, integrating the method with machine learning algorithm such as RFC could be done to determine the factors that greatly contribute to the relationship considered.

### 5.2 Practical implication

With the exponential increase of Samgyeopsal restaurants in the Philippines and with the surge of branches opening, this study was able to deduce that hedonic and utilitarian motivation, attitude, and Korean influence led to very high actual behavior to eat in Samgyeopsal restaurants in the Philippines. The feeling of happiness and enjoyment, pleasant environment, and functionality should be taken into consideration by restaurateurs. The setting of ambiance to Korean culture, playing of Korean music, and continuous offering of unlimited food at an affordable price may be capitalized on. Business strategies could be created with these indicators to promote the consumption of Samgyeopsal. Moreover, creating promotions for mass events and gatherings may be done right after the COVID-19 pandemic to enhance profitability and marketing.

### 5.3 Limitations

Despite the strength of the findings, there are still limitations being considered in this study. First, the study considered collecting the responses through a self-administered survey online. Further results and factors may be evident if an interview was conducted along with the survey. Second, this study was conducted during the COVID-19 pandemic. The results may be different when the respondents were able to consume Samgyeopsal with no restrictions set by the pandemic. In addition, another perspective of the findings would be evident if the distribution of demographic characteristics were identified. Due to the strict lockdown implementation, the study was only able to collect the data utilizing an online survey. Lastly, further classification using conjoint analysis and K-Means clustering for market segmentation may be done to classify the respondents of this study. To which, business strategies and target consumers may be highlighted.

## 6. Conclusion

The increase of Samgyeopsal restaurants has become evident in the past years with a lot of branches opening in different areas of the Philippines. This study aimed to determine the factors of affection intention to eat Samgyeopsal in the Philippines during the COVID-19 pandemic. Specifically, this study considered extending the TPB and analyzed using SEM and RFC to classify the different factors affecting actual behavior to consume Samgyeopsal.

Based on the RFC results, hedonic and utilitarian motivation, Korean influence, and attitude led to very high actual behavior. Moreover, SEM also indicated that the constructs of TPB and the COVID-19 safety protocol were significant factors that affected the actual behavior to eat Samgyeopsal in the Philippines. Business strategies may be created based on the indicators presented in the result of this study. The results of this study would be beneficial to Korean BBQ restaurateurs and the further development of their marketing strategies even in other countries. Finally, the model construct of this study can be extended and applied in evaluating the consumers’ eating intentions toward other varieties of food or cuisines worldwide.
